# Lipid Analysis of the 6-Hydroxydopamine-Treated SH-SY5Y Cell Model for Parkinson’s Disease

**DOI:** 10.1007/s12035-019-01733-3

**Published:** 2019-09-06

**Authors:** Helena Xicoy, Jos F. Brouwers, Oleksandra Kalnytska, Bé Wieringa, Gerard J. M. Martens

**Affiliations:** 1 Department of Cell Biology, Radboud Institute for Molecular Life Sciences, Radboud University Medical Centre, Geert Grooteplein Zuid 26-28, 6525 GA Nijmegen, The Netherlands; 2 Department of Molecular Animal Physiology, Faculty of Science, Donders Institute for Brain, Cognition and Behaviour, Donders Centre for Neuroscience, Geert Grooteplein Zuid 26-28, 6525 GA Nijmegen, The Netherlands; 3 Department of Biochemistry & Cell Biology, Lipidomics Facility, Faculty of Veterinary Medicine, Utrecht University, Yalelaan 2, 3584 CM Utrecht, The Netherlands

**Keywords:** Parkinson’s disease, Lipidomics, Phospholipids, Sphingomyelin, Cholesterol, SH-SY5Y cells

## Abstract

Parkinson’s disease (PD) is a highly prevalent neurodegenerative disease for which no disease-modifying treatments are available, mainly because knowledge about its pathogenic mechanism is still incomplete. Recently, a key role for lipids emerged, but lipid profiling of brain samples from human subjects is demanding. Here, we used an unbiased approach, lipidomics, to determine PD-linked changes in the lipid profile of a well-established cell model for PD, the catecholaminergic neuronal cell line SH-SY5Y treated with the neurotoxin 6-hydroxydopamine (6-OHDA). We observed changes in multiple lipid classes, including phosphatidylcholine (PC), phosphatidylglycerol (PG), phosphatidylinositol (PI), phosphatidylserine (PS), sphingomyelin (SM), and total cholesterol, in 6-OHDA-treated SH-SY5Y cells. Furthermore, we found differences in the length and degree of unsaturation of the fatty acyl chains, indicating changes in their metabolism. Except for the observed decreased PS levels, the alterations in PC, PG, PI, and cholesterol levels are in agreement with the results of previous studies on PD-patient material. Opposite to what has been previously described, the cholesterol-lowering drug statins did not have a protective effect, while low doses of cholesterol supplementation partially protected SH-SY5Y cells from 6-OHDA toxicity. However, cholesterol supplementation triggered neuronal differentiation, which could have confounded the results of cholesterol modulation. Taken together, our results show that 6-OHDA-treated SH-SY5Y cells display many lipid changes also found in PD patient and animal model brains, although the SH-SY5Y cell model seems less suitable to study the involvement of cholesterol in PD initiation and progression.

**Electronic supplementary material:**

The online version of this article (10.1007/s12035-019-01733-3) contains supplementary material, which is available to authorized users.

## Background

Parkinson’s disease (PD) is the second most common neurodegenerative disease with a prevalence of around 9.5 per 1000 people aged at least 65–70 years [1]. PD presents with motor and non-motor symptoms [2, 3] that worsen with advancing age, leading to a need for assistance with all daily activities. The main pathological hallmarks of PD are progressive loss of dopaminergic neurons in the substantia nigra (SN) projecting to the striatum, formation of Lewy bodies (abnormal protein aggregates containing α-synuclein), and microgliosis (activated microglia) [4]. However, the molecular mechanisms underlying these neuropathological features are currently not fully understood.

In order to better discern the molecular mechanisms involved in the initiation and progression of PD, we have recently built a so-called molecular landscape for PD based on genetic information from the familial forms of PD (which account for 10% of the cases) and data from genome-wide association studies (GWAS) on sporadic PD patients [5]. This unbiased, hypothesis-free approach did not only corroborate evidence regarding the pathways that are thought to play a role in PD but also provided novel insight, in particular regarding the key role of lipids in PD etiology. Abnormal lipid composition of cellular membranes is known to affect α-synuclein aggregation, mitophagy, and immune responses, i.e., processes linked to PD etiology [6–8]. Other studies have revealed that dietary intake of cholesterol and polyunsaturated fatty acids (PUFAs) is associated with PD [9–11], and that omega-3 PUFAs seem to have a beneficial role in dopaminergic neurons [12, 13].

On the basis of these observations and predictions, we hypothesized that defects in lipid composition and metabolism may contribute to the etiology of PD, and that lipids and lipid-metabolizing enzymes could constitute targets for therapy or modulation of the disease [14]. Since studies on the initiation and progression of PD in humans are difficult, the catecholaminergic neuroblastoma cell line SH-SY5Y treated with the catecholaminergic neurotoxin 6-hydroxydopamine (6-OHDA) has been widely used as a model to mimic PD [15–17]. Although multiple other studies have already been performed to establish the value of 6-OHDA-treated SH-SY5Y cells as PD-model [18], the resemblance between the brain and serum lipidomes of PD patients and the 6-OHDA-induced lipid profile of SH-SY5Y cells has—to our knowledge—not been investigated until now. Therefore, we decided to perform an in vitro study to explore the effect of 6-OHDA on the lipidome of SH-SY5Y cells. This unbiased lipidomics approach allowed us to profile differences in lipid species between SH-SY5Y cells treated at two time points with various doses of 6-OHDA.

## Methods

### Cell Culture Conditions

The SH-SY5Y human neuroblastoma cell line (ATCC® CRL-2266™) expressed the neuronal marker β-III tubulin (Online resource 1a), the catecholaminergic marker l-3,4-dihydroxyphenylalanine (l-DOPA, Online resource 1b), and the dopaminergic marker tyrosine hydroxylase (TH, Online resource 1c). The cells were grown in modified Dulbecco’s Eagle media with 10% fetal bovine serum, 1% antibiotic-antimycotics, 1% GlutaMAX, and 1% sodium pyruvate, and incubated in 5% CO_2_ at 37 °C. Cells were kept no longer than passage 20 after acquisition. Cells were detached from the culture vessel using a short treatment with trypsin-EDTA and passaged once a week after they reached 80% confluency. All media components and the trypsin-EDTA were from Thermo Fisher Scientific (Gibco™).

### 6-OHDA Treatment

Cells were seeded in 6-well plates at a density of 2.5 × 10^5^cells/mL. After 24 h of adherent growth, cells were treated for 12 or 24 h with different concentrations of 6-OHDA (Sigma). The chemical compound 6-OHDA mimics PD because it is able to enter neurons through dopaminergic or noradrenergic transporters and trigger reactive oxygen species formation and oxidative stress [15]. For RNA analysis, the cells were washed with phosphate-buffered saline (PBS) and taken up by direct detachment in Trizol Reagent (Sigma). For lipidomic analysis, the cells were washed with cold PBS, detached with trypsin-EDTA, resuspended in cold PBS, and centrifuged, and the pellets were snap frozen in liquid nitrogen and stored at − 80 °C until use.

### Lipidomics

Pellets of cultured cells were mixed with UPLC-grade chloroform: methanol 1:1 (v/v) and, after 20 min, samples were centrifuged at 2000×*g* and the supernatant was used directly for LC-MS analysis. To this end, 10 μL was injected on a hydrophilic interaction liquid chromatography (HILIC) column (2.6 μm HILIC 100 Å, 50 × 4.6 mm, Phenomenex, Torrance, CA) and eluted with a gradient from ACN/Acetone (9:1, v/v) to ACN/H2O (7:3, v/v) with 10 mM ammonium formate, and both with 0.1% formic acid. Flow rate was 1 mL/min. The column outlet of the LC was either connected to a heated electrospray ionization source of a LTQ-XL mass spectrometer or a Fusion mass spectrometer (both from ThermoFisher Scientific, Waltham, MA). Full-scan spectra were collected from *m*/*z* 450–950 at a scan speed of 3 scans/s in both positive- and negative ionization mode (LTQ-XL). On the Fusion, full spectra were collected in negative ionization mode from *m*/*z* 400 to 1600 at a resolution of 120,000. Parallel data-dependent MS2 was done in the linear ion trap at 30% HCD collision energy. During lipid extraction and storage, a nitrogen atmosphere was maintained to prevent lipid peroxidation. The absence of oxysterols in the analysis of sterols illustrated that lipid peroxidation had not occurred [19].

### Cholesterol

Cholesterol was measured essentially as described previously [20]. In brief, extracted lipids were eluted from a RP-HPLC column with a gradient of MeOH:2-propanol (8:2, v/v) in MeOH:H2O (1:1, v/v) from a 2 × 150 mm HALO-C18 column (Advanced Materials Technology, Wilmington, DE). Cholesterol was measured by monitoring the transition from *m*/*z* 369.3, corresponding to [M+H-H2O]+, to its most abundant fragment at *m*/*z* 161.1. A response factor was calculated using an external calibration curve.

For data analysis, data were converted to mzXML or mzML format and analyzed using XCMS version 1.52.0 running under R version 3.4.3 (R Development Core Team: A language and environment for statistical computing, 2016. URL http://www.R-project.org). Carbon-13 de-isotoping and identification of lipid species was done in R by matching MS signals to lipid classes based on retention time and molecular species were subsequently assigned based on *m*/*z* matching to an in silico generated lipid MS database*.*

### RNA Isolation

For RNA isolation, the Trizol cell mixtures were resupended and incubated for 25 min at 4 °C, followed by incubation for 5 min at room temperature in Eppendorf tubes. Then, RNase-free chloroform (80 μL) was added and tubes were shaken and briefly vortexed. Next, samples were incubated for 2–3 min at RT and centrifuged for 15 min. The aqueous phase was recovered and 1 μL of glycogen carrier (20 μg/mL) was added to each sample. Samples were vortexed shortly before adding 200 μL of isopropanol. Samples were mixed by inversion, incubated for 10 min at RT and centrifuged for 10 min. Supernatant was removed and 0.5 mL of ice-cold (− 20 °C) 75% ethanol was added to the pellet before a very short vortex and 5 min of centrifugation. Again, the supernatant was removed and the washing step with ice-cold ethanol was repeated. Next, supernatant was completely removed and the pellet was air-dried for 10–15 min, dissolved in 15 μL of autoclaved Milli-Q water, and incubated for 10 min at 55 °C before storing the samples at − 80 °C. The samples were kept at 4 °C at all steps (including centrifugation), except otherwise indicated.

### cDNA Synthesis and QRT-PCR Quantification

Total cDNA was synthesized from 1 μg of total RNA following the instructions of the ReverseAid First Strand cDNA synthesis kit (Fermentas Life Sciences). The 30 μL of cDNA obtained were diluted with 420 μL of Milli-Q water, and stored at 4 °C. Quantitative real-time PCR was performed using SYBRGreen (Bioline). Briefly, reaction mixtures consisting of 5 μL of SYBRGreen, 1.8 μL of water, 0.6 μL of forward primer, 0.6 μL of reverse primer, and 2 μL of cDNA mixture were assembled for each sample (Table 1 for primer sequences). Segments from each of the different cDNAs were PCR amplified with the following program: 2 min at 95 °C, 40 cycles of 95 °C for 5 s, 65 °C for 10 s, and 72 °C for 15 s, and a gradient from 70 to 95 °C; on a Rotor Gene Q series. The quantification was accomplished by considering both the takeoff and amplification values of each sample and using a normalization value obtained from the housekeeping genes GAPDH and YWHAZ with the GeNorm2 algorithm [21].

**Table 1:** List of primers used to identify changes in mRNA levels of lipid-related genes

Gene	Forwards primer	Reverse primer
DHCR7	GCCGGTTCAAGAAGGAAAAGT	AGATGCGGTTCTGTCATTGGT
GAPDH (housekeeping gene)	ACCACCCTGTTGCTGTAGCC	GACTTCAACAGCGACACCCA
HMGCR	TTGGCAGCAGGACATCTTGTC	AGAACCCAATGCCCATGTTC
LRP1	GACGCAGCTCAAGTGTGCCC	TGGCCATCTGTTCCACGTGG
SREBF1	AGCCAGCCTGACCATCTGTGA	GCACGGCCTTGTCAATGGAG
YWHAZ (housekeeping gene)	CCAACACATCCTATCAGACTGGG	TCAGCAATGGCTTCATCAAAAG

### Cholesterol and Simvastatin Treatment

SH-SY5Y cells were plated in 96-wells plates (for a cell survival assay) or seeded in 8-well ibidi plates (ibidi, Cat. no: 80826) (for immunocytochemistry or live-cell imaging) at a density of 2.5 × 10^5^cells/mL. After 24 h, the cells were treated with 2.5 to 40 μM SyntheChol™ Supplement (Sigma) or 10 nM to 5 μM of the cholesterol-lowering drug simvastatin (Sigma), and/or 25 μM 6-OHDA. After 24 h, cells were washed with PBS and fixed with either cold 10% trichloroacetic acid (Sigma) or 4% paraformaldehyde for cell survival assay or immunocytochemical analysis, respectively.

### Cell Survival Analysis (Sulforhodamine B, SRB, Assay)

After fixation with cold (4 °C) 10% trichloroacetic acid for 1 h at 4 °C, cells were washed three times with Milli-Q water. After removing the last wash, 0.5% SRB solution was added to each well. Cells were incubated in SRB for 15 min at RT in the dark and washed four times with 1% acetic acid (Sigma). All acetic acid was tapped out the plate after the last washing step and the plate was dried at 60 °C for 10–15 min. Finally, 150 μL of 10 mM Tris-HCl (pH = 10) (ICN) was added to each well, its contents mixed to homogeneity, and the OD of the colored solution was measured with a Biorad plate reader at 510 nm.

### Immunocytochemistry

Ibidi-well (ibidi, Cat. no: 80826) adherent cells were fixed with 4% paraformaldehyde for 20 min at RT and washed three times with PBS. Thereafter, cells were incubated with blocking buffer for 1 h at RT and incubated with the primary antibody (mouse β-III tubulin, 1:100 (Covance); rabbit anti-tyrosine hydroxylase, 1:1000 (Pel-Freez Biologicals); rabbit anti-l-dopa, 1:1000 (Abcam)) in blocking solution overnight at 4 °C. Next, the preparations were washed three times with PBS and incubated with the secondary antibody (Alexa Fluor 568 goat against mouse, 1:500 (Life Technologies); Alexa Fluor 488 goat against rabbit, 1:500 (Life Technologies)) and DAPI (1:500) diluted in blocking buffer for 2 h at RT, in the dark. Finally, cells were washed three times with PBS and pictures were taken with an automated high-content microscope (DMI6000B, Leica). Imaging was performed with the × 20 objective; autofocus was set on the DAPI signal at every position, with a local focus of 30 μm and medium precision. Image analysis was performed with ImageJ, whereby β-III tubulin intensity was normalized for the number of cells in each image. Per condition, at least 9 images with a minimum of 200 cells per image were quantified in each of the triplicates of the experiment.

### Live-Cell Imaging

Cells were seeded in 12-well plates at a density of 2.5 × 10^5^cells/mL. After 24 h of adherent growth, CellEvent™ Caspase-3/7 Green ReadyProbes™ Reagent (Thermo Fisher Scientific) was added to cells immediately upon treatment with 40 μM of cholesterol and/or 25 μM 6-OHDA, and imaging was performed for 24 h with a Zeiss Axiovert 200M microscope with Moticam-pro 2850 CCD Camera, Okolab stage incubator and run by Micromanager 1.4 software.

### Statistical Analysis

Grouped data are expressed as mean ± SD and individual values are plotted. Changes between groups were analyzed by two-way ANOVA and Dunnett correction for multiple comparisons using statistical hypothesis testing (adjusted *p* value) using Graphpad Prism (San Diego, CA). All measurements were repeated at least three times. Adjusted *p* value < 0.05 was accepted as significant.

## Results

### Lipidomic Analysis of 6-OHDA-Treated SH-SY5Y Cells

**Fig. 1:**
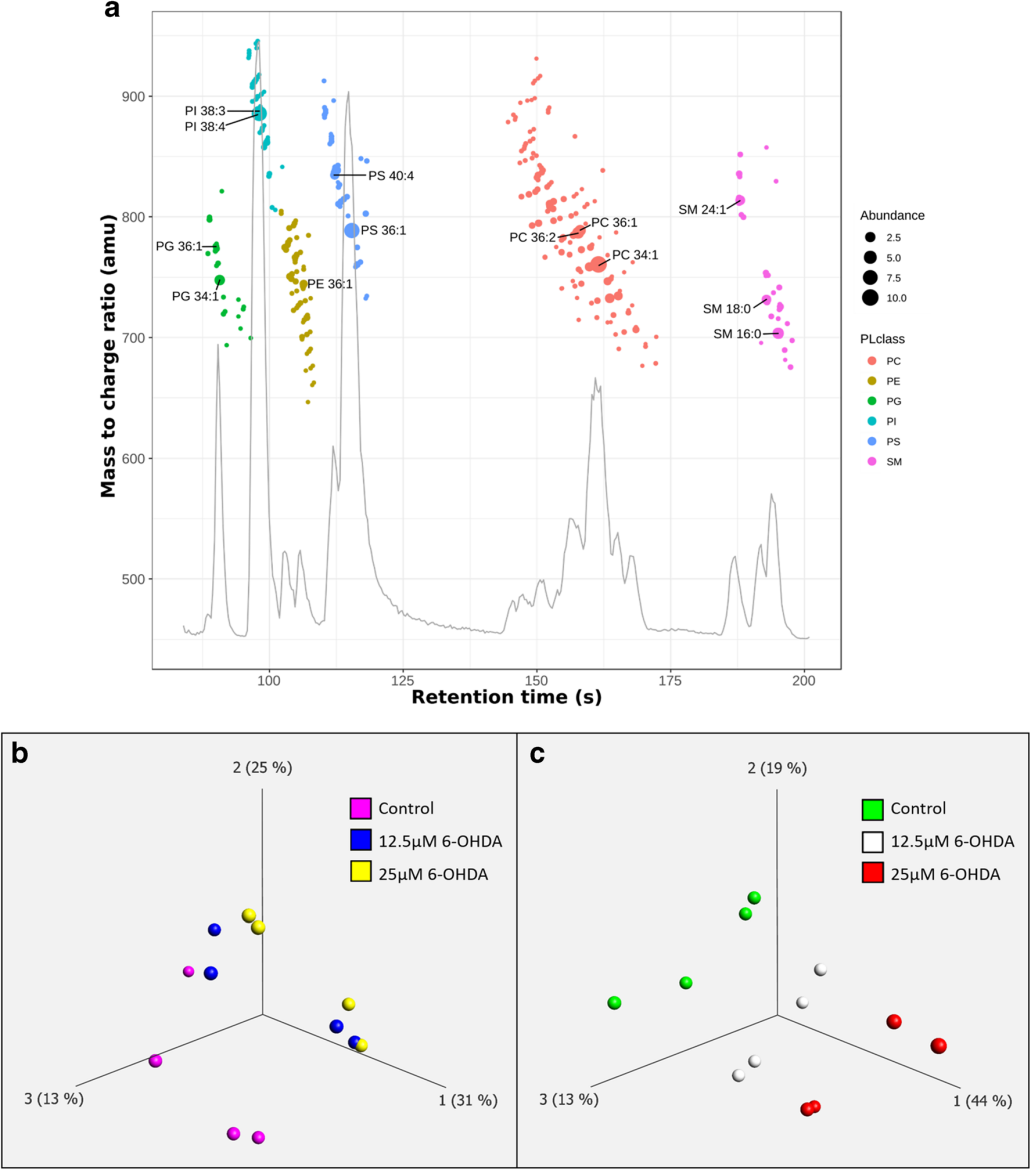
Lipidomic analysis of 6-OHDA-treated SH-SY5Y cells. **a** Base peak chromatogram of the separation of phospholipid classes. Detected molecular species are plotted as an overlay. Abundance of each lipid specie is represented by the size of the dot. Each lipid class corresponds to a color. **b** Three-dimensional principal component analysis (PCA) plot including all three conditions at 12 h and **c** 24 h. Dots with the same color represent four biological replicates. The plot reduces the dimensionality of the data by projecting the 216 variance-filtered lipids into three principal components (axes 1, 2, and 3). The percentage of variation explained by each principal component is specified between brackets

We first analyzed the changes in the lipid profile of SH-SY5Y cells treated with 6-OHDA. To avoid confounding effects, we chose for cell treatments with 12.5 μM and 25 μM 6-OHDA for 12 and 24 h, i.e., conditions under which the maximum induction of apoptosis was expected to be 50% (Online resource 2). The lipid composition of the cells was analyzed by LC-MS. We identified 306 phospholipids from the following classes: phosphatidylcholine (PC), phosphatidylethanolamine (PE), phosphatidylglycerol (PG), phosphatidylinositol (PI), and phosphatidylserine (PS), and the sphingolipid sphingomyelin (SM). After filtering for variance, a total of 216 lipids were kept for further analysis (Online resource 3). A three-dimensional principal component analysis (PCA) plot of these lipids shows that at 12 h, the control condition is different from the two 6-OHDA treatments, while the two treatments do not greatly differ from one another (Fig. 1a). A similar plot at 24 h shows a clear difference between the three treatment groups (Fig. 1b).

#### Global Changes

**Fig. 2:**
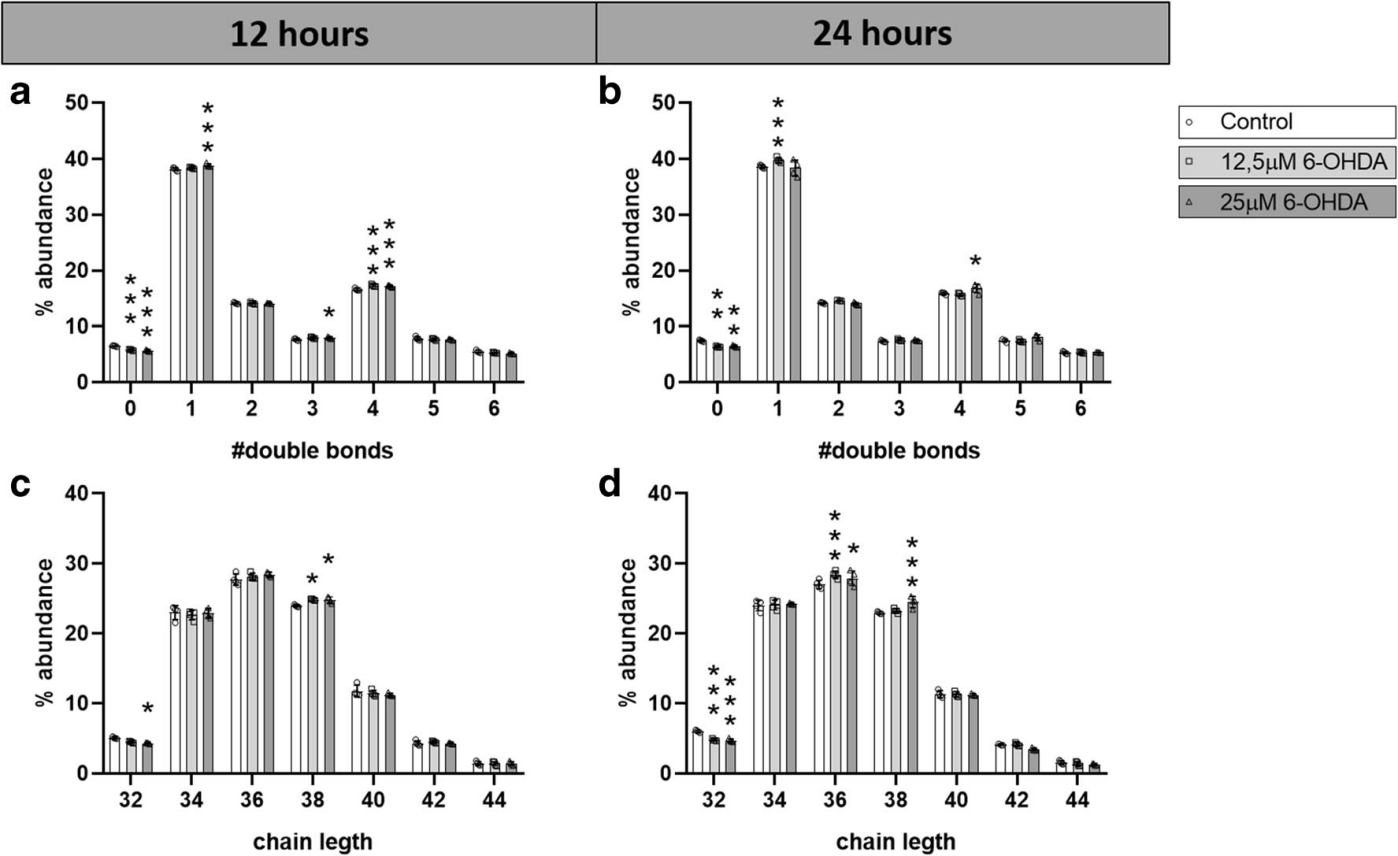
Double bonds and carbon chain length of fatty acyl chains. Distribution of double bonds in fatty acyl chains after **a** 12 h and **b** 24 h of treatment; distribution of carbon chain length after **c** 12 h and **d** 24 h of treatment. *N* = 4. Dunnett corrected *p* values of differences relative to the control. *p < 0.05; **p < 0.01; ***p < 0.001

We observed that a 12-h treatment with 12.5 μM 6-OHDA increased the levels of fatty acyl chains with 4 double bonds and decreased levels of those without double bonds (Fig. 2a), while 25 μM significantly increased the levels of side chains with 1, 3, and 4 double bonds, and decreased the levels of those without double bonds (Fig. 2a). After 24 h of treatment, 12.5 μM 6-OHDA increased the levels of fatty acyl side chains with 1 double bond and decreased those without double bonds, while treatment with 25 μM 6-OHDA significantly increased the levels of side chains with 4 double bond, and decreased those without double bonds (Fig. 2b). These findings suggest that 6-OHDA interferes with the process of lipid unsaturation, decreasing fatty acyl side chains without double bonds in most conditions, and increasing those with 4 double bonds.

Furthermore, the 12-h 12.5 μM 6-OHDA treatment resulted in a significant increase in the number of 38 carbons-fatty acyl side chains. Similarly, the 12-h 25 μM 6-OHDA treatment led to a significant increase in the levels of lipids with 38 carbon-fatty acyl side chains and a decrease in those with 32 carbons (Fig. 2c). The 24-h treatment with 12.5 μM 6-OHDA significantly decreased the amounts of lipids with 32 carbons-fatty acyl side chains and increased those with 36 carbons, while 25 μM 6-OHDA significantly increased the levels of lipids with fatty acyl side chains of 36 and 38 carbons in length, and decreased those with 32 carbons in length (Fig. 2d). Therefore, the average abundance of each fatty acyl chain length in the total analysis was also modified by 6-OHDA treatment, with a global trend to a decrease of fatty acyl side chains with 32 carbons and an increase of those with 36 and 38 carbons.

#### Changes in PC

**Fig. 3:**
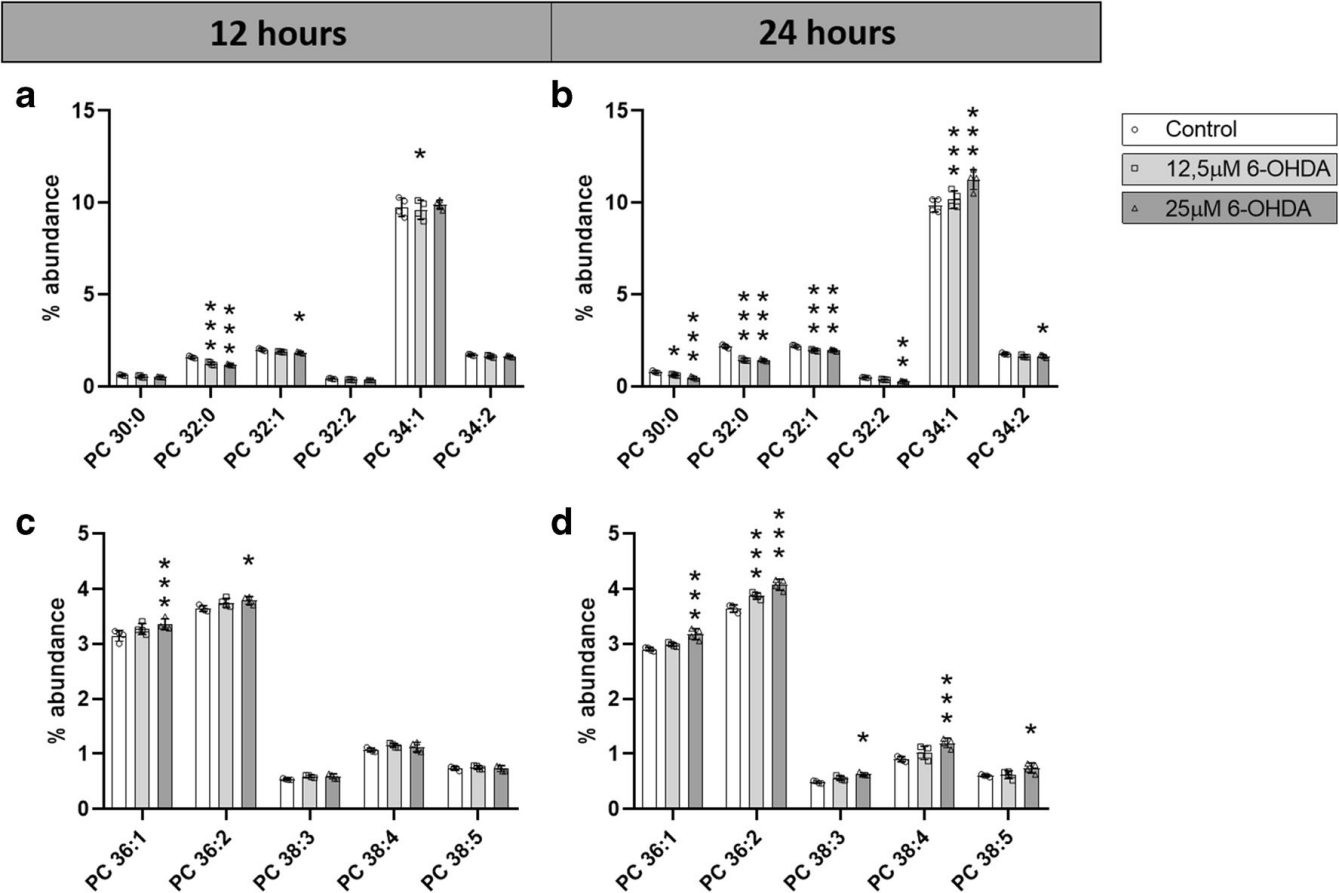
Abundance of individual PC species. Percentage of abundance of individual PC species after **a**, **c** 12 h and **b**, **d** 24 h of treatment with 0 μM, 12.5 μM, or 25 μM 6-OHDA. *N* = 4. Dunnett corrected *p* values of differences relative to the control. *p < 0.05; **p < 0.01; ***p < 0.001

Eleven out of 72 individual PC species showed a significant change in at least one condition, and only PC 32:0 was significantly reduced in all four conditions. Interestingly, PC species with shorter fatty acyl chains (30 and 32) were decreased in 6-OHDA-treated SH-SY5Y cells, while those with longer fatty acyl side chains (36 and 38) were significantly increased (Fig. 3a–d). Only PC 34:1 was discordant in the direction of changes between the time points, namely downregulated at 12 h upon treatment with 12.5 μM 6-OHDA and upregulated at 24 h with the 12.5 μM and 25 μM 6-OHDA treatments.

#### Changes in PG

**Fig. 4:**
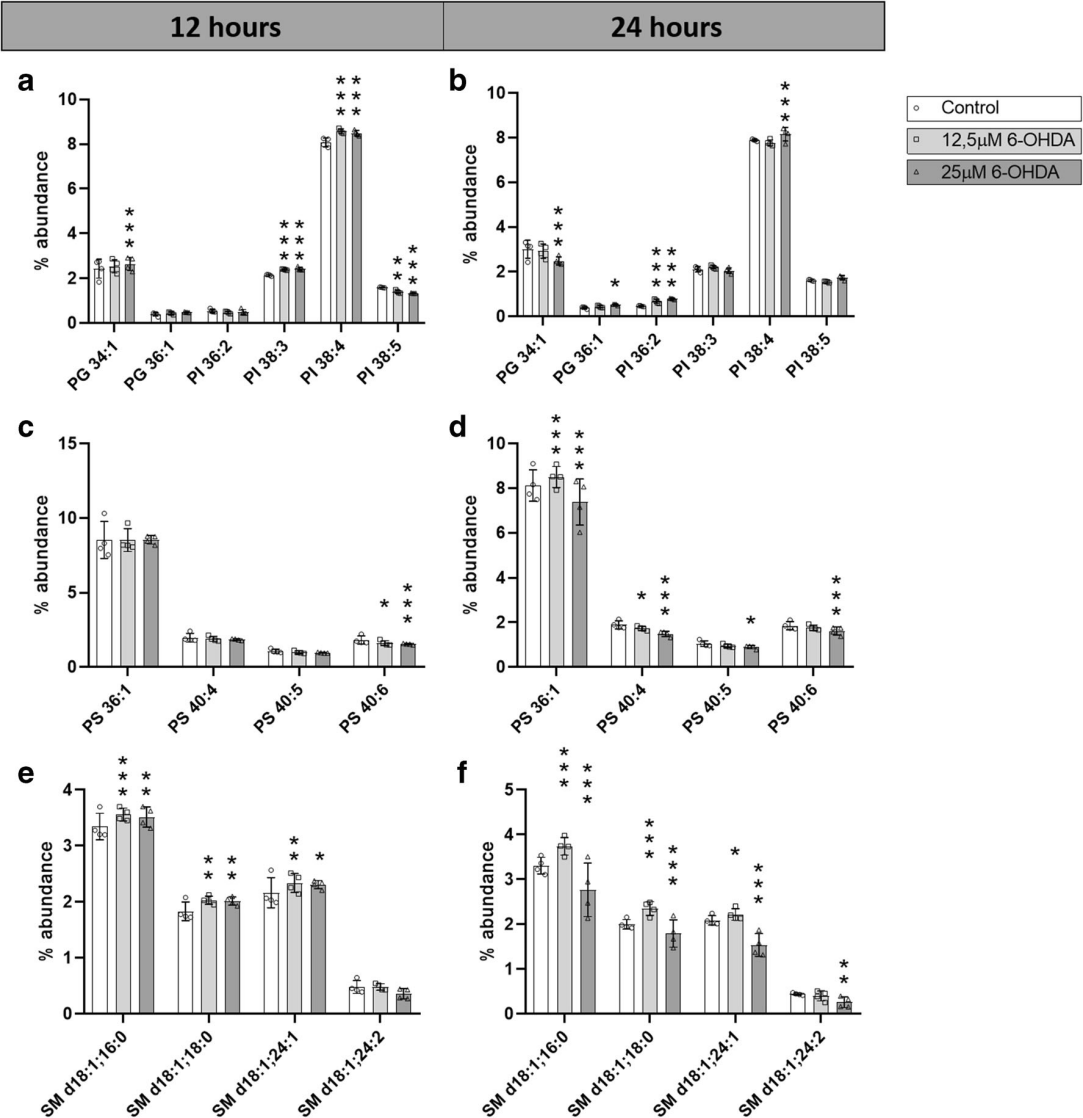
Abundance of specific PG, PI, PS, and SM species. Percentage of abundance of individual PG and PI species after **a** 12 h and **b** 24 h of treatment with 0 μM, 12.5 μM, or 25 μM 6-OHDA. Percentage of abundance of individual PS species after **c** 12 h and **d** 24 h of treatment with 0 μM, 12.5 μM, or 25 μM 6-OHDA. Percentage of abundance of individual SM species after **e** 12 h and **f** 24 h of treatment with 0 μM, 12.5 μM, or 25 μM 6-OHDA *N* = 4. Dunnett corrected *p* values of differences relative to the control. *p < 0.05; **p < 0.01; ***p < 0.001

Two out of 15 individual PG species showed a significant change in at least one condition. More specifically, PG 34:1 was increased after 12 h of treatment with 25 μM 6-OHDA, while it was significantly decreased after 24 h of the same treatment, and PG 36:1 was increased in cells treated with 25 μM 6-OHDA for 24 h (Fig. 4a, b).

#### Changes in PI

PC and PG showed larger differences after 24 than 12 h of treatment, while PI mainly changed at 12 h of treatment (Fig. 4a, b). Four out of 27 PI species showed a significant change in at least one condition. The differences in PI 38:3, 38:4, and 38:5 were statistically significant after 12 h of treatment, from which the first two species increased in treated cells, while the last one decreased. Only PI 36:2 was increased after 24 h of treatment with both 12.5 μM and 25 μM 6-OHDA, while the abundance of PI 38:4 increased in SH-SY5Y cells treated with 25 μM 6-OHDA for 24 h.

#### Changes in PS

All changes in the abundance of individual PS species were reductions. A total of four species out of the 34 identified, namely PS 36:1, 40:4, 40:5, and 40:6, were downregulated at least in one of the treatment conditions (Fig. 4c, d). Remarkably, PS 40:6 was significantly decreased under three out of the four conditions.

#### Changes in SM

Four out of 29 individual SM species showed a significant change in at least one condition. Remarkably, all changes at 12 h and those after 24 h of 12.5 μM 6-OHDA increased in concentration, while the differences of cells treated with 25 μM 6-OHDA for 24 h decreased (Fig. 4e, f).

### 6-OHDA Treatment Changes Cholesterol Levels in SH-SY5Y Cells

**Fig. 5:**
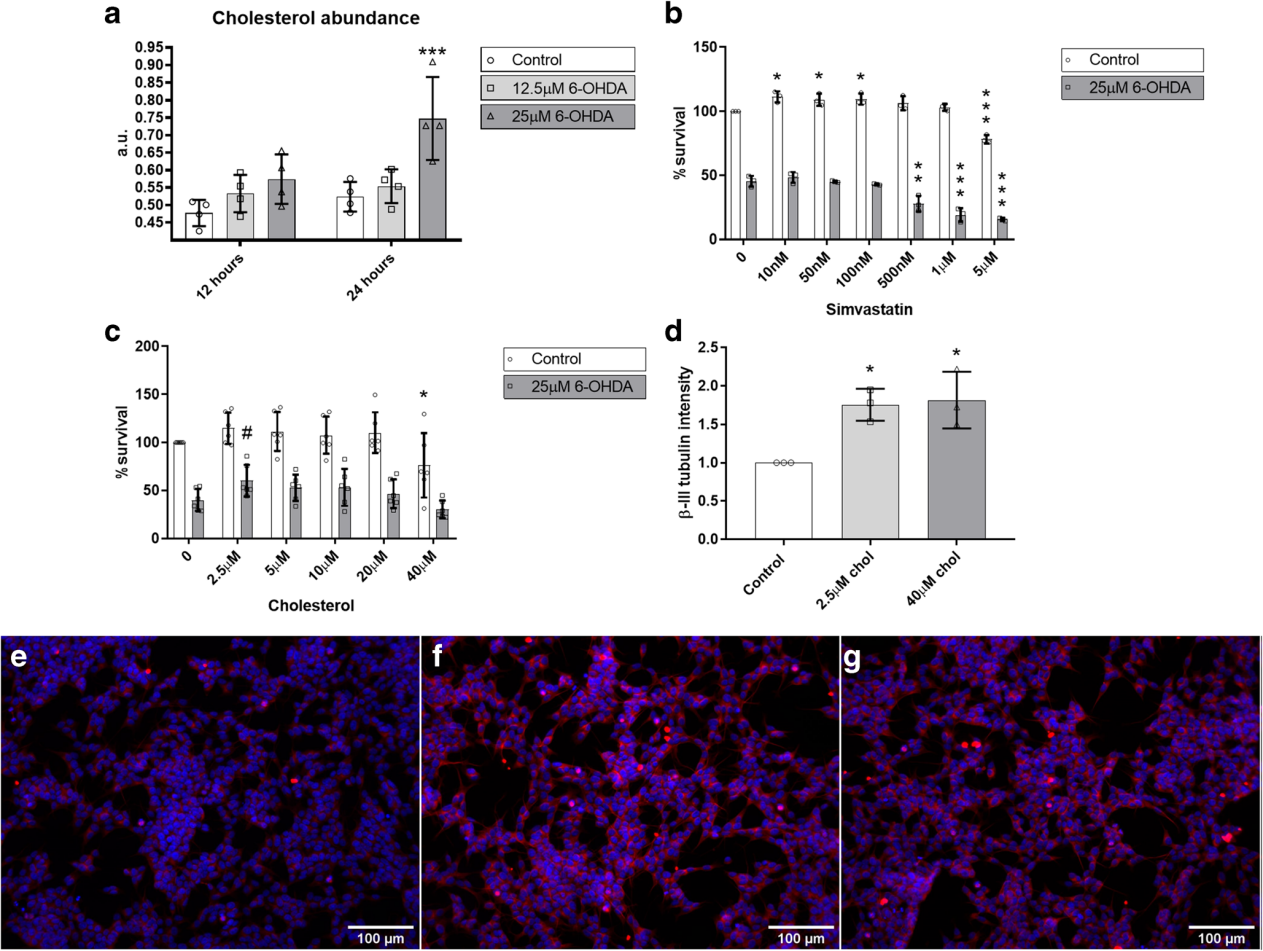
Effects of cholesterol manipulation. **a** Changes in cholesterol abundance in SH-SY5Y cells after a 12-h or 24-h treatment with 0 μM, 12.5 μM, or 25 μM 6-OHDA, in arbitrary units (a.u.). *N* = 4. **b** Effect of 10 nM to 5 μM simvastatin treatment on cell survival of SH-SY5Y cells treated with (6-OHDA) or without (control) 6-OHDA. *N* = 3. **c** Effect of 2.5 μM to 40 μM cholesterol treatment on cell survival of SH-SY5Y cells treated with (6-OHDA) or without (control) 6-OHDA. *N* = 4. **d** Intensity of β-III tubulin staining on SH-SY5Y cells treated with 0 μM, 2.5 μM, or 40 μM of cholesterol. Differences relative to the control. Representative images of SH-SY5Y cells treated with **e** 0 μM, **f** 2.5 μM, or **g** 40 μM cholesterol, stained with DAPI (blue) and β-III tubulin (red). *N* = 4. ^#^*p* < 0.1 (trend); *p < 0.05; **p < 0.01; ***p < 0.001

Since multiple studies point to the involvement of cholesterol changes in PD, we analyzed the total cholesterol abundance in SH-SY5Y cells treated with 0 μM, 12.5 μM, and 25 μM 6-OHDA for 12 or 24 h. The treatment with 12.5 μM 6-OHDA did not change cholesterol abundance in SH-SY5Y cells. However, we observed that SH-SY5Y cells treated with 25 μM 6-OHDA for 12 h showed a trend towards increased cholesterol levels (adjusted *p* value, 0.0909), while a 24-h treatment showed a statistically significant increase in cholesterol abundance compared to the control situation (Fig. 5a).

Additionally, we compared the differential effects of 12- and 24-h treatment of SH-SY5Y cells with 0, 12.5, and 25 μM 6-OHDA on the expression levels of mRNAs for enzymes/receptors involved in cholesterol metabolism, namely SREBF1, DHCR7, HMCGR, and LRP1. In an attempt to discriminate between apoptosis-inducing and genuine PD-related effects of 6-OHDA, we also analyzed the effects of etoposide, an apoptosis inducer that produces double-strand breaks by forming a ternary complex with DNA and topoisomerase II, leading to cell death unrelated to PD. Again, to avoid confounding effects, we chose for a cell treatment with 5 μM etoposide for 24 h, conditions under which the induction of apoptosis was around 50% (Online resource 2). The three concentrations of 6-OHDA significantly decreased the mRNA expression of SREBF1 and DHCR7 after 24 h of treatment, while there were no significant changes in HMGCR and LRP1 mRNA levels at any time point or concentration (Online resource 4). Etoposide treatment decreased the expression of SREBF1 and DHCR7 mRNAs both at 12 and 24 h, increased the expression of LRP1 mRNA only at 24 h, and did not alter the level of HMGCR mRNA (Online resource 4). Hence, the changes in mRNA expression of cholesterol-associated genes are similar in 6-OHDA- and etoposide-treated cells, suggesting an apoptosis-like signature.

### A Low Dose of Cholesterol, but Not Simvastatin, Reduces 6-OHDA Toxicity in SH-SY5Y Cells

Since SH-SY5Y cells treated with 25 μM 6-OHDA for 24 h displayed increased cholesterol levels and to determine if we could rescue 6-OHDA toxicity, we tried to compensate the increase in cholesterol by culturing the 6-OHDA-treated and untreated cells in the presence of various concentrations of the cholesterol-lowering drug simvastatin. At low doses (10-100 nM), simvastatin slightly increased SH-SY5Y cell proliferation compared to untreated cells, whereas a dose of 5 μM simvastatin was detrimental. On the other hand, we did not observe any improvement of SH-SY5Y cell survival upon culturing the 6-OHDA-treated cells in the presence of a low dose of simvastatin, while concentrations ranging from 500 nM to 5 μM simvastatin enhanced 6-OHDA toxicity (Fig. 5b).

Since the use of a cholesterol-lowering drug did not protect 6-OHDA-treated SH-SY5Y cells, we hypothesized that cells expressing higher cholesterol levels are more protected against 6-OHDA treatment. Thus, increased cholesterol levels may be beneficial. We therefore treated SH-SY5Y cells with media containing various doses of cholesterol (2.5 to 40 μM) and 25 μM 6-OHDA for 24 h. A low dose of cholesterol (2.5 μM) showed a trend towards increased survival after 6-OHDA treatment (*p* = 0.058), while high doses of cholesterol (40 μM) had detrimental effects on cell survival (*p* = 0.0269) (Fig. 5c).

We tried to confirm high-dose cholesterol toxicity with a time-lapse of the cells treated with 40 μM cholesterol and/or 25 μM 6-OHDA, and an early caspase indicator. We did not observe increased cell death in cells treated with only 40 μM cholesterol, but cells treated with 40 μM cholesterol and 25 μM 6-OHDA died earlier than cells treated with only 25 μM 6-OHDA (Online resource 5). However, cells supplemented with 40 μM cholesterol seemed to proliferate less, which, as previously described [22, 23], could be due to the occurrence of SH-SY5Y cell differentiation. Using β-III tubulin as a marker for neuronal differentiation, SH-SY5Y cells treated with 0 μM, 2.5 μM, or 40 μM cholesterol for 24 h displayed a significant increase in β-III tubulin protein expression (Fig. 5d). This finding indicates that in this model, cholesterol may have effects beyond neuroprotection, e.g., on neuronal differentiation.

## Discussion

In the present study, we performed a lipidomic analysis of SH-SY5Y cells treated with 6-OHDA, a broadly used cell model to study aspects of PD pathology. We observed that 6-OHDA leads to increased levels of unsaturated lipids (i.e., a decrease in lipid species without double bonds, and an increase in species with one or four double bonds). Interestingly, monounsaturated fatty acids, which have one double bond, are involved in α-synuclein toxicity, and inhibition of the rate-limiting enzyme in their synthesis, stearoyl CoA desaturase, leads to increased lipid species without double bonds and is protective in various cellular and animal PD models [24, 25]. However, decreased unsaturation indices have been described in lipid rafts from the frontal cortex of PD patients [26]. We also found that 6-OHDA treatment results in an increase of lipids with longer fatty acyl side chains (36 and 38 carbons in length). A SNP in *ELOVL7*, a member of the elongase family that plays a role in the elongation of acyl-CoA with a C18 carbon chain length [27], has been associated with PD in a meta-analysis of GWAS results from over 20,000 PD cases and almost 400,000 controls of European ancestry [28], and to early-, but not late-onset PD in a GWAS on a Chinese population [29]. A defect in *ELOVL7* leads to an accumulation of its substrate and could result in increased C36 and C38 phospholipids, in line with our observations and thus supporting the use of 6-OHDA-treated SH-SY5Y cells as an early-PD model.

In the 6-OHDA-treated SH-SY5Y cells, we observed both decreased and increased levels of PC, depending on their fatty acyl side chain length. The phospholipid PC is the most abundant in mammalian membranes [30] and it is involved in neuronal differentiation, neurite outgrowth, and axonal elongation [31]. When looking at individual species, those with shorter fatty acyl side chains, namely PC 30:0, 32:0, 32:1, 32:2, and 34:2, were decreased in 6-OHDA-treated cells. Decreased levels of PC 34:2 have also been described in plasma of PD patients [32]. Similarly, decreased levels of overall PC have been described in the SN of male PD patients [33], in the SN of rats infused with 6-OHDA [34], and in goldfish treated with the PD-linked neurotoxin prodrug 1-methyl-4-phenyl-1,2,3,6-tetrahydropyridine [35]. However, PC with longer fatty acyl side chains, including PC 36:1, 36:2, 38:3, 38:4, and 38:5, were increased in 6-OHDA-treated cells. Interestingly, one of the enzymes involved in PC synthesis, namely phosphocholine cytidylyltransferase, is elevated in the SN of PD patients [36].

We detected increased levels of PI 38:3 and 38:4. PI is not only a constituent of the cell membrane, but its metabolites are also involved in processes such as cell signaling, vesicular trafficking, metabolism, and pre- and post-synapse formation [37–39]. A trend towards increased PI has also been observed in skin fibroblasts from parkin-mutant PD patients [32] and in lipid rafts from the frontal cortex of early-PD patients [26]. Moreover, PI enhances α-synuclein association with the membrane and increases its self-interactivity and self-oligomerization [40, 41]. Hence, our results are in line with the observed increase of PI in PD, which might be linked to α-synuclein toxicity.

We found a decrease in the levels of four species of PS, the main anionic phospholipid in the plasma membrane of neural tissue, which plays both a structural and a signaling role in the cell [42]. Decreased PS levels, as observed in our PD-cell model, have also been reported in plasma from PD patients [32]. However, increased PS levels have been found in brains of aged (male) mice overexpressing α-synuclein [43], skin fibroblasts from parkin-mutant PD patients [44], and elevated activity of phosphatidylserine synthase (the enzyme responsible for PS synthesis) has been found in the SN of PD patients [36].

In our cell model, we observed an increase in three SM species at 12 h, persisting after 24 h of treatment with the lowest concentration of 6-OHDA, while the 25 μM 6-OHDA treatment led to a decrease of these species at 24 h. SM is the most abundant sphingolipid in eukaryotic cells and, in the nervous system, it is the main constituent of myelin. *SMPD1* encodes sphingomyelin phosphodiesterase, and mutations in this gene result in SM accumulation and are a risk factor for PD [45–47]. Additionally, increased levels of SM have been observed in the primary visual cortex and SN of PD patients [33, 48].

Besides the changes in PC, PG, PI, PS, and SM lipid classes, it is of note that five lipid species, including PC 34:1, PG 34:1, and SM d18:1;16:0, which are likely to have the same side chain lipid composition with an oleic acid (18:1) and a palmitic acid (16:0), are discordant between time points and/or concentrations. An interconversion between these lipids may be relevant to the disease. For example, the synthesis of SM from PC and ceramide, catalyzed by sphingomyelin synthase, has been shown to be involved in the production and release of endosomes [49], which can trigger dopaminergic neurodegeneration [50]. Hence, the implications of these discordant changes remain to be established.

Finally, we observed increased levels of cholesterol in the 6-OHDA-treated SH-SY5Y cells. The human brain harbors around 25% of the cholesterol present in the human body, which needs to be mainly synthesized in situ, since most plasma proteins carrying cholesterol cannot cross the blood-brain barrier [51, 52]. Cholesterol is involved in cell membrane fluidity and the formation of lipid rafts, which play a role in growth factor signaling, axon guidance, and synaptic formation, among others [51]. Our results are consistent with the increased cholesterol levels observed in the visual cortex of early-PD patients [48]. Furthermore, cholesterol has been linked to lysosomal and endosomal dysfunction as well as to α-synuclein aggregation [51, 53, 54] and has been demonstrated to contribute to dopaminergic neuronal loss in a mouse model for PD [55]. Total cholesterol levels also represent a PD-risk factor [56] and the use of cholesterol-lowering drugs, statins, has been associated with multiple mechanisms that may improve dopaminergic neuronal survival, although its impact is controversial (reviewed in [57]). Moreover, total cholesterol levels appear to be genetically associated with PD [5].

Since we do not observe any improvement on survival of 6-OHDA-treated cells upon simvastatin treatment, but an increase in cell death at high doses of this lipid-lowering drug, our results are not in line with its antiapoptotic effect [58], but support a role of statins in SH-SY5Y apoptosis through the mitochondrial pathway [59, 60]. However, part of the neuroprotective or neurorestorative effects of statins has been attributed to an increase of presynaptic dopaminergic biomarkers [61] and thus possibly induction of a dopaminergic phenotype, which could have a detrimental effect on a dopaminergic cell toxicity model such as 6-OHDA-treated SH-SY5Y cells. Therefore, it would be interesting to study the effects of various statins on the differentiation of SH-SY5Y cells in order to clarify the here found increased sensitivity of these cells to 6-OHDA.

On the other hand, we observed a trend towards a protective role of low doses of cholesterol in the 6-OHDA-treated SH-SY5Y cells and found exacerbated toxicity of 6-OHDA at high doses of cholesterol, which is in agreement with previous studies using SH-SY5Y cells treated with 24- and 27-hydroxycholesterol, which are the cholesterol metabolites that can cross the blood-brain barrier [62, 63]. Cholesterol treatment was also found to increase neuronal differentiation, which is in line with previous investigations [22, 23]. Therefore, our results concerning the effects of increased cholesterol in the PD-cell model support previous observations. However, one should realize that SH-SY5Y cell studies in which cholesterol levels are modulated might give ambiguous results due to its effect on cell differentiation.

## Conclusions

In conclusion, the alterations in the lipid profile of SH-SY5Y cells treated with low doses of 6-OHDA for 12 and 24 h appear to mimic a substantial number of lipid changes that have been also reported for the brain lipidome of (early) PD patients and PD mouse models. Our findings thus support the validity of cultured 6-OHDA-treated SH-SY5Y cells as an attractive cell model for in vitro studies on PD. However, one has to bear in mind that a single clonal cell type does not fully mimic the complex changes that occur in PD patient material.

## Electronic Supplementary Material

Online resource 1 Immunocytochemical characterization of SH-SY5Y cells. Immunocytochemistry of SH-SY5Y cells with DAPI (blue) and the neuronal marker β-III tubulin (a), the catecholaminergic marker L-dopa (b), and the dopaminergic marker tyrosine hydroxylase (TH, c). (PNG 1700 kb)

Online resource 2 Dose-response of SH-SY5Y cells treated with 6-OHDA or etoposide. Percentages of cells surviving the treatment with (a) 0 μM (control), 12.5 μM, 25 μM, 50 μM and 100 μM 6-OHDA for 24 h, or (b) 0 μM (control), 5 μM, 10 μM, 20 μM and 40 μM etoposide for 24 h. *N* = 3. (PNG 27 kb)

Online resource 3 Lipidomic analysis of SH-SY5Y cells treated with 0 μM, 12.5 μM and 25 μM 6-OHDA for 12 and 24 h. List of all lipids detected together with their percentages of abundance and summary statistics on annotated lipids. (XLSX 63 kb)

Online resource 4 mRNA expression of cholesterol-related genes in SH-SY5Y cells treated with 6-OHDA and etoposide. Relative mRNA expression levels of markers of cholesterol metabolism and transport (SREBF1, HMCGR, DHCR7 and LRP1) on SH-SY5Y cells treated with 6-OHDA (0 μM, 12.5 μM and 25 μM) or etoposide (5 μM). N = 3–6. FDR corrected *p*-values. *p < 0.05; **p < 0.01, ***p < 0.001. (PNG 131 kb)

Online resource 5 Live-cell imaging of SH-SY5Y cells treated with 6-OHDA and/or cholesterol. SH-SY5Y cells were treated with 25 μM of 6-OHDA and/or 40 μM of cholesterol and monitored by time-lapse imaging for 24 h. Cells were cultured in the presence of CellEvent™ Caspase-3/7 Green ReadyProbes™ Reagent to detect apoptosis. (AVI 27732 kb)

## Abbreviations

6-OHDA: 6-hydroxydopamine
GWAS: genome-wide association studies
PC: phosphatidylcholine
PD: Parkinson’s disease
PE: phosphatidylethanolamine
PI: phosphatidylinositol
PS: phosphatidylserine
SM: sphingomyelin
SN: substantia nigra
SRB: sulforhodamine
