# Supplementary figures and images for: Lipid Analysis of the 6-Hydroxydopamine-Treated SH-SY5Y Cell Model for Parkinson’s Disease
Source: Mol Neurobiol. 2019 Sep 6;57(2):848–59. doi: 10.1007/s12035-019-01733-3 (PMC7031185; doi:10.1007/s12035-019-01733-3)

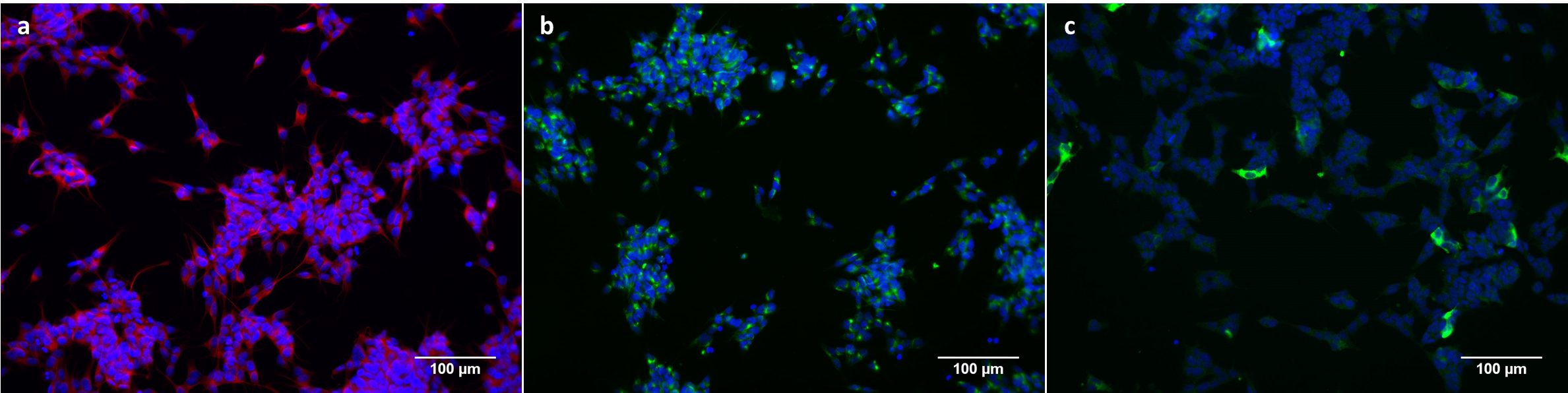

Supplement: Supplementary file 1 — Immunocytochemical characterization of SH-SY5Y cells. Immunocytochemistry of SH-SY5Y cells with DAPI (blue) and the neuronal marker β-III tubulin (a), the catecholaminergic marker L-dopa (b), and the dopaminergic marker tyrosine hydroxylase (TH, c). (PNG 1700 kb) [file 12035_2019_1733_MOESM1_ESM.png]

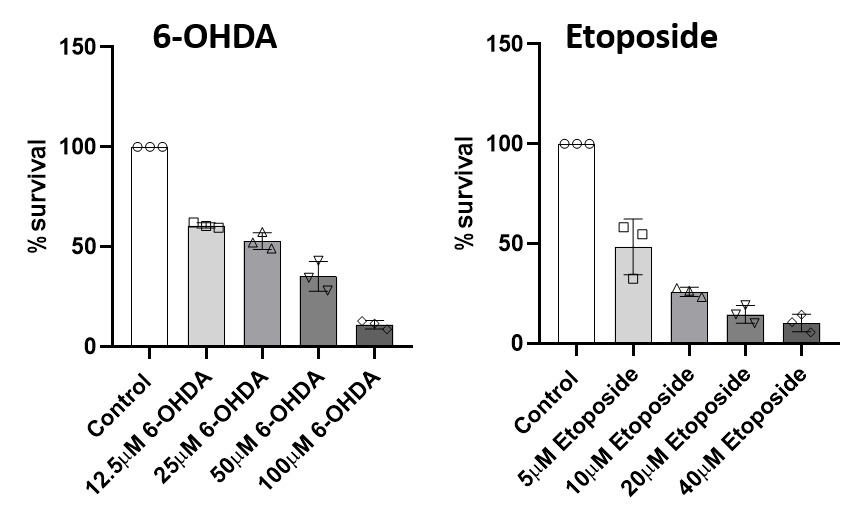

Supplement: Supplementary file 2 — Dose-response of SH-SY5Y cells treated with 6-OHDA or etoposide. Percentages of cells surviving the treatment with (a) 0 μM (control), 12.5 μM, 25 μM, 50 μM and 100 μM 6-OHDA for 24 h, or (b) 0 μM (control), 5 μM, 10 μM, 20 μM and 40 μM etoposide for 24 h. N = 3. (PNG 27 kb) [file 12035_2019_1733_MOESM2_ESM.png]

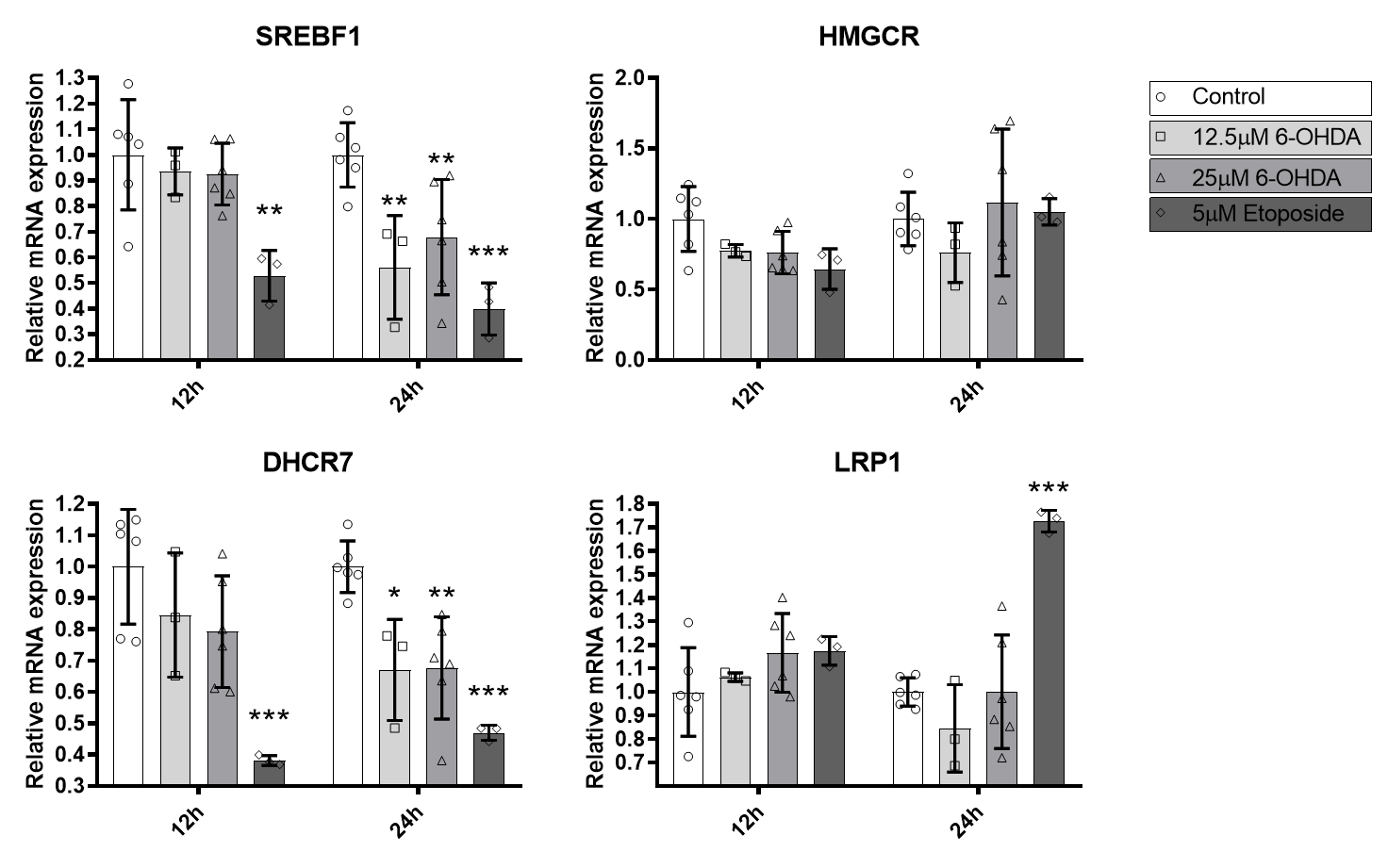

Supplement: Supplementary file 4 — mRNA expression of cholesterol-related genes in SH-SY5Y cells treated with 6-OHDA and etoposide. Relative mRNA expression levels of markers of cholesterol metabolism and transport (SREBF1, HMCGR, DHCR7 and LRP1) on SH-SY5Y cells treated with 6-OHDA (0 μM, 12.5 μM and 25 μM) or etoposide (5 μM). N = 3–6. FDR corrected p-values. *p < 0.05; **p < 0.01, ***p < 0.001. (PNG 131 kb) [file 12035_2019_1733_MOESM4_ESM.png]
